# Left atrial appendage occlusion in the absence of intraprocedural product specialist monitoring: is it time to proceed alone? Results from a multicenter real-world experience

**DOI:** 10.3389/fcvm.2023.1172005

**Published:** 2023-06-13

**Authors:** Davide Margonato, Vincenzo Rizza, Giacomo Ingallina, Alberto Preda, Francesco Ancona, Martina Belli, Cosmo Godino, Eustachio Agricola, Paolo Della Bella, Carmelo Grasso, Marco Contarini, Patrizio Mazzone

**Affiliations:** ^1^ Department of Cardiovascular Imaging, IRCCS San Raffaele Hospital and Vita-Salute University, Milan, Italy; ^2^ Department of Cardiac Electrophysiology and Arrhythmology, IRCCS San Raffaele Hospital and Vita-Salute University, Milan, Italy; ^3^ Department of Clinical Cardiology, IRCCS San Raffaele Hospital and Vita-Salute University, Milan, Italy; ^4^ Department of Cardiology, C.A.S.T., Azienda Ospedaliero Universitaria Policlinico “G. Rodolico-San Marco”, University of Catania, Catania, Italy; ^5^ Department of Interventional Cardiology, Umberto I Hospital, Syracuse, Italy

**Keywords:** left atrial appendage closure, intraprocedural monitoring, cardiovascular mortality, embolic prevention, atrial fibrillation

## Abstract

**Background:**

Percutaneous left atrial appendage occlusion (LAAO) presents many technical complex features, and it is often performed under the intraprocedural surveillance of a product specialist (PS). Our aim is to assess whether LAAO is equally safe and effective when performed in high-volume centers without PS support.

**Methods:**

Intraprocedural results and long-term outcome were retrospectively assessed in 247 patients who underwent LAAO without intraprocedural PS monitoring between January 2013 and January 2022 at three different hospitals. This cohort was then matched to a population who underwent LAAO with PS surveillance. The primary end point was all-cause mortality at 1 year. The secondary end point was a composite of cardiovascular mortality plus nonfatal ischemic stroke occurrence at 1 year.

**Results:**

Of the 247 study patients, procedural success was achieved in 243 patients (98.4%), with only 1 (0.4%) intraprocedural death. After matching, we did not identify any significant difference between the two groups in terms of procedural time (70 ± 19 min vs. 81 ± 30 min, *p* = 0.106), procedural success (98.4% vs. 96.7%, *p* = 0.242), and procedure-related ischemic stroke (0.8% vs. 1.2%, *p* = 0.653). Compared to the matched cohort, a significant higher dosage of contrast was used during procedures without specialist supervision (98 ± 19 vs. 43 ± 21, *p* < 0.001), but this was not associated with a higher postprocedural acute kidney injury occurrence (0.8% vs. 0.4%, *p* = 0.56). At 1 year, the primary and the secondary endpoints occurred in 21 (9%) and 11 (4%) of our cohort, respectively. Kaplan–Meier curves showed no significant difference in both primary (*p* = 0.85) and secondary (*p* = 0.74) endpoint occurrence according to intraprocedural PS monitoring.

**Conclusions:**

Our results show that LAAO, despite the absence of intraprocedural PS monitoring, remains a long-term safe and effective procedure, when performed in high-volume centers.

## Introduction

The growing need for the use of left atrial appendage occlusion (LAAO) devices is a direct consequence of the call for stroke prevention in many patients with atrial fibrillation (AF) either not eligible for or not compliant with oral anticoagulation therapies (1). Evidence has recently emerged, both from randomized clinical trials and registries, confirming the long-term safety and efficacy of LAA closure devices as a strategy for thromboembolic prevention in patients with AF (2–4).

However, this procedure requires a structured training process, as the operator must be able to become independent in handling many complex features of this procedure: the transeptal puncture, the highly variable anatomy of the LAA, the specific characteristics of each different device, and the potential complications such as cardiac tamponade and device embolization (5, 6). Therefore, many centers perform LAA closure under the surveillance of the product specialist (PS) from the device manufacturer. Recent COVID-19 pandemic has raised the issue of limited access of nonhospital staff to operative rooms, and, particularly in this setting, we performed different procedures without the help of the PS. We believe that in centers with high volumes, procedures, and experienced operators (both interventionalist and echocardiographers) who have a deep understanding of the respective imaging methods, i.e., fluoroscopy and transesophageal echocardiography (TEE) (7), an optimal result may be achieved without the intraprocedural assistance of the PS. Therefore, the purpose of this study is to present our experience on the feasibility and the short- and long-term safety and efficacy of the LAA closure procedure performed without the intraprocedural assistance of the PS.

## Methods

### Study design

This is a multicenter retrospective study carried out at three different Italian centers. We searched in our clinical databases for AF patients who underwent percutaneous LAAO at our centers from January 2013 to January 2022. Patients were considered eligible if, during this period, LAAO was performed in the absence of intraprocedural support by the PS and if 1-year follow-up after the day of the procedure was available. Data were recorded in a dedicated database in compliance with the ethic committees of our centers. All patients provided informed consent before the procedure.

### Patient population management

Baseline clinical characteristics and therapy were recorded for all the patients. TEE was routinely performed before procedure by a senior echocardiographer using a Vivid E95 (GE HealthCare, United States) with a 6VT-D probe. The LAAO procedure was performed as previously described (8, 9), directly by or under the supervision of the same operator (PM, MC, and CG). The devices used were the Watchman (Boston Scientific, St. Paul, MN, United States), with different generations of occluders related to the year of the procedure. To minimize the risk of complications, intraprocedural TEE monitoring was always conducted, and cerebral protection devices were used according to the operator's discretion. Before the release of the device, its position, anchoring, and sizing were evaluated, and procedure success was defined, after device release, in the absence of all the following: pericardial effusion causing hemodynamic instability, device embolization, procedure-related stroke, or significant paradevice leak (PDL, >5 mm at a Nyquist limit of 20–30 cm/s^7^). As for the peri- and postprocedural thromboembolic complications, ischemic stroke was defined as a sudden onset of a focal or global neurological deficit, lasting >24 h or <24 h, but with imaging-documented new or presumed new ischemic lesion (10); transient ischemic attack (TIA) was defined as a neurological dysfunction lasting <24 h and without any new alteration identified on imaging studies (10). Finally, major bleeding was defined as type III or V of the Bleeding Academic Research Consortium classification (11).

### Outcome

The primary end point was all-cause death (ACD) at 1 year. The secondary end point was a composite of cardiovascular (CV) mortality (defined as death due to advanced heart failure, coronary ischemic heart disease, sudden cardiac death, or due to stroke) plus nonfatal ischemic stroke occurrence at 1 year.

Clinical follow-up was performed with routine visits according to our internal protocol and via phone contact.

### Statistical analyses

Categorical variables were expressed as count (percentage) and compared with the *χ*^2^ or Fisher exact test. Continuous variables were expressed as mean (SD) or median [interquartile range (IQR)], and Student's *t* and ANOVA tests were used as appropriate.

Survival and event-free survival were estimated by the Kaplan–Meier (KM) method and compared by log-rank test. Analysis was performed by censoring follow-up at time of last follow-up or at the time of event occurred. A two-tailed *p* <0.05 was considered statistically significant.

To confirm the feasibility and both short- and long-term safety and efficacy of LAAO performed in the absence of the PS, this group was matched 1:1 to patients who underwent LAAO in our center to prevent ischemic events with the intraprocedural supervision of the PS and performed by the same above-mentioned interventionalists. To reduce the potential for imbalance in baseline characteristics among the two groups, a propensity score matching with the use of 1:1 nearest-neighbor strategy was used. The baseline characteristics matched among the two groups are the following: age, gender, hypertension, diabetes mellitus, chronic kidney disease (CKD, defined as eGFR < 60 ml/min/m^2^), CHA2DS2-VASc score, and a history of ischemic stroke or hemorrhagic stroke. After matching, *p* values <0.05 were considered statistically significant. Statistical analysis was performed in the R environment (R Foundation for Statistical Computing, Vienna, Austria).

## Results

### Study population

Out of the 1,097 patients treated with LAAO in the designated period, 321 (29%) procedures were performed in the absence of intraprocedural PS monitoring: clinical follow-up after procedure up to 1 year was available in 247 patients, therefore representing the population included in our study. Baseline characteristics of the population are shown in Table 1. Patients were predominantly male, with multiple cardiovascular risk factors and comorbidities, particularly CKD and history of previous cerebrovascular events. The clinical profile was well balanced between ischemic and hemorrhagic risk. Most of them were treated with a single anticoagulant therapy (60%) at the time of the procedure, and LAAO was mainly performed because of a high hemorrhagic risk or a previous major bleeding (79%). Procedures were most commonly performed during the COVID-19 pandemic (from March 2020, 154 patients) compared to the pre-COVID period (93 patients).

**Table 1:** Baseline clinical characteristics (247 patients).

Variable	
Age	76 ± 4
Gender (female, %)	94 (38%)
BMI (kg/m^2^)	27 ± 4
Smoking (either former or current)	109 (44%)
Comorbidities	
Dislipidemia	163 (66%)
Hypertension	193 (78%)
Diabetes mellitus	72 (29%)
Chronic heart failure	51 (21%)
Carotid vasculopathy	30 (12%)
Peripheral artery disease	54 (22%)
DCM	35 (14%)
CKD	138 (56%)
Previous ischemic stroke	42 (17%)
Previous hemorrhagic stroke	35 (14%)
Previous TIA	20 (8%)
Previous CABG	22 (9%)
AF	247 (100%)
Paroxysmal	99 (40%)
Persistent	30 (12%)
Permanent	118 (48%)
Previous AF ablation	12 (5%)
Risk scores	
CHA2DS2-VASc score, median ± IQR	4 ± 2
HAS-BLED, median ± IQR	4 ± 1
Antithrombotic treatment (pre-LAAO)	
SAPT	30 (12%)
Aspirin	22 (9%)
P2Y12 inhibitors	8 (3%)
Single anticoagulant therapy	148 (60%)
LWMH	71 (29%)
Warfarin	20 (8%)
DOAC	57 (23%)
DAPT	12 (5%)
Dual antithrombotic therapy	22 (9%)
Aspirin + LWMH	6 (2%)
Aspirin + DOAC	7 (3%)
P2Y12 inhibitors + DOAC	9 (4%)
Triple antithrombotic therapy	35 (14%)
DAPT + LWMH	25 (10%)
DAPT + DOAC	10 (4%)
DOAC at reduced dose according to Guidelines (8)	32 (13%)
Echocardiographic parameters	
LVEF (%)	48 ± 10
LAVi (ml/m^2^)	72 ± 20
LAA morphology	
Chicken wing	84 (34%)
Windsock	45 (18%)
Cauliflower	81 (33%)
Cactus	37 (15%)
Reason for LAAO	
Patient's choice	7 (3%)
Thrombosis during anticoagulant therapy	25 (10%)
Bleeding during anticoagulant therapy	12 (5%)
High hemorrhagic risk	94 (38%)
Previous major bleeding	104 (41%)
Unstable INR	5 (3%)

BMI, body mass index; DCM, dilated cardiomyopathy; CABG, coronary artery bypass graft; CV, cardiovascular; CKD, chronic kidney disease; AF, atrial fibrillation; PM, pacemaker; ICD, implantable cardioverter defibrillator; CRT, cardiac resynchronization therapy; TIA: transient ischemic attack; LAAO, left atrial appendage occlusion; SAPT, single anti-platelet therapy; DAPT, dual anti-platelet therapy; LWMH, low-weight molecular heparin; DOAC, direct oral anticoagulant; VKA, vitamin-k antagonist; LAVi, Left atrial volume indexed; LVEF, left ventricular ejection fraction; MV, mitral valve; AV, aortic valve; INR, international normalized ratio.

Values are mean ± SD or *n* (%), unless otherwise specified.

### Intraprocedural outcomes

**Figure 1:**
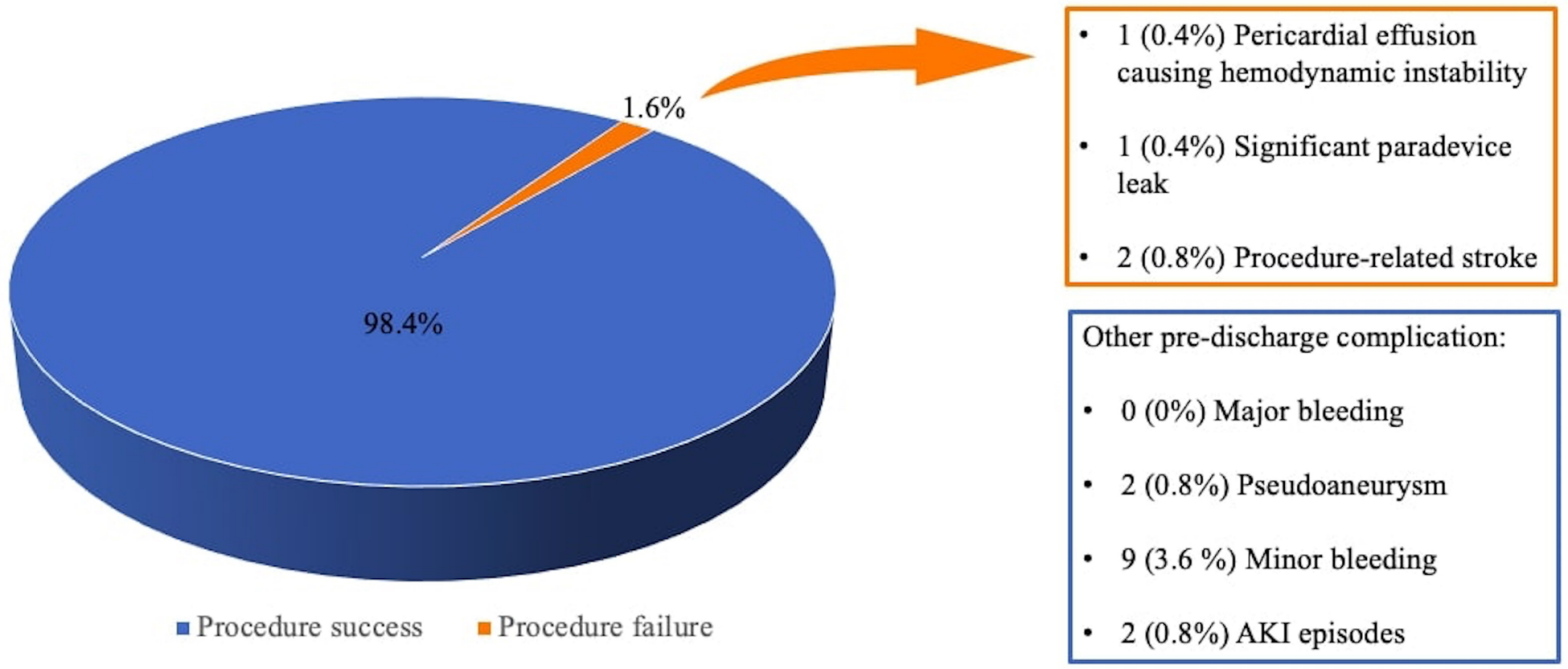
Procedure-related outcome and events.

Out of 247 LAAO interventions, procedure success was achieved in 243 patients (98.4%, see Figure 1), with only one cardiac tamponade leading to cardiac arrest and death; intraoperative echocardiographic evaluation revealed one significant paradevice leak and two patients suffered from procedure-related ischemic stroke. Average procedure time was 70 ± 19 min with a mean contrast dosage of 98 ± 19 ml. Periprocedural complications consisted of vascular access site pseudoaneurysm, acute kidney injury (AKI), and minor bleeding (see Figure 1).

### Matched cohort and procedural outcome

Propensity score matching matched 494 patients 1:1 between LAAO without intraprocedural PS supervision (*n* = 247) and LAA closure with intraprocedural support (*n* = 247) based on similar propensity scores. Baseline characteristics of propensity-matched pairs stratified by different intraprocedural LAAO approach were almost identical (see Table 2).

**Table 2:** Two groups comparison after propensity score matching.

	LAAO without product specialist supervision (*n* = 247)	Matched cohort (*n* = 247)	*p*-value
Age (years)	76 ± 4	77 ± 5	0.354
Gender (female)	94 (38%)	87 (35%)	0.575
Hypertension	193 (78%)	195 (79%)	0.828
Diabetes mellitus	72 (29%)	93 (37%)	0.05
CKD	138 (56%)	150 (61%)	0.202
CHA2DS2-VASc score	4 ± 2	4 ± 6	0.064
Previous ischemic stroke	42 (17%)	43 (17%)	0.905
Previous hemorrhagic stroke	35 (14%)	33 (13%)	0.794

LAAO, left atrial appendage occlusion; CKD, chronic kidney disease.

After matching, we did not identify any significant difference between the two populations in terms of procedural time, cardiac tamponade causing hemodynamic instability, intraprocedural leak, or procedure-related stroke, as shown in Table 3. A significant higher dosage of contrast media was used during procedures without specialist supervision (98 ± 19 ml vs. 43 ± 21 ml, *p* < 0.001), but this was not associated with a higher postprocedural AKI occurrence (*p* = 0.56); the mean Mehran score for those who developed AKI was 15 ± 1, implying a risk of any contrast-induced nephropathy of 26.1% (12). Compared to the matched cohort, minor bleedings were more common in the cohort of patients without intraprocedural PS support (9 episodes vs. 2, *p* = 0.033), while the number of major bleeding episodes was significantly inferior (0 episodes vs. 4, *p* = 0.045).

**Table 3:** Differences in procedure-related events according to intraprocedural presence of product specialist.

Parameter	LAAO without product specialist supervision (*n* = 247)	Matched cohort with intraprocedural supervision (*n* = 247)	*p*-value
Procedural time (min)	70 ± 19	81 ± 30	0.106
Contrast dosage (ml)	98 ± 19	43 ± 21	<0.001
Procedural success	243 (98.4%)	239 (96.7%)	0.242
Intraprocedural leak	1 (0.4%)	3 (1.2%)	0.315
Pericardial effusion with hemodynamic instability	1 (0.4%)	2 (0.8%)	0.56
Procedure-related ischemic stroke	2 (0.8%)	3 (1.2%)	0.653
Major bleeding	0 (0%)	4 (1.6%)	0.045
Minor bleeding	9 (3.6%)	2 (0.8%)	0.033
AKI	2 (0.8%)	1 (0.4%)	0.56

LAAO, left atrial appendage occlusion; AKI, acute kidney injury.

### Outcome

**Figure 2:**
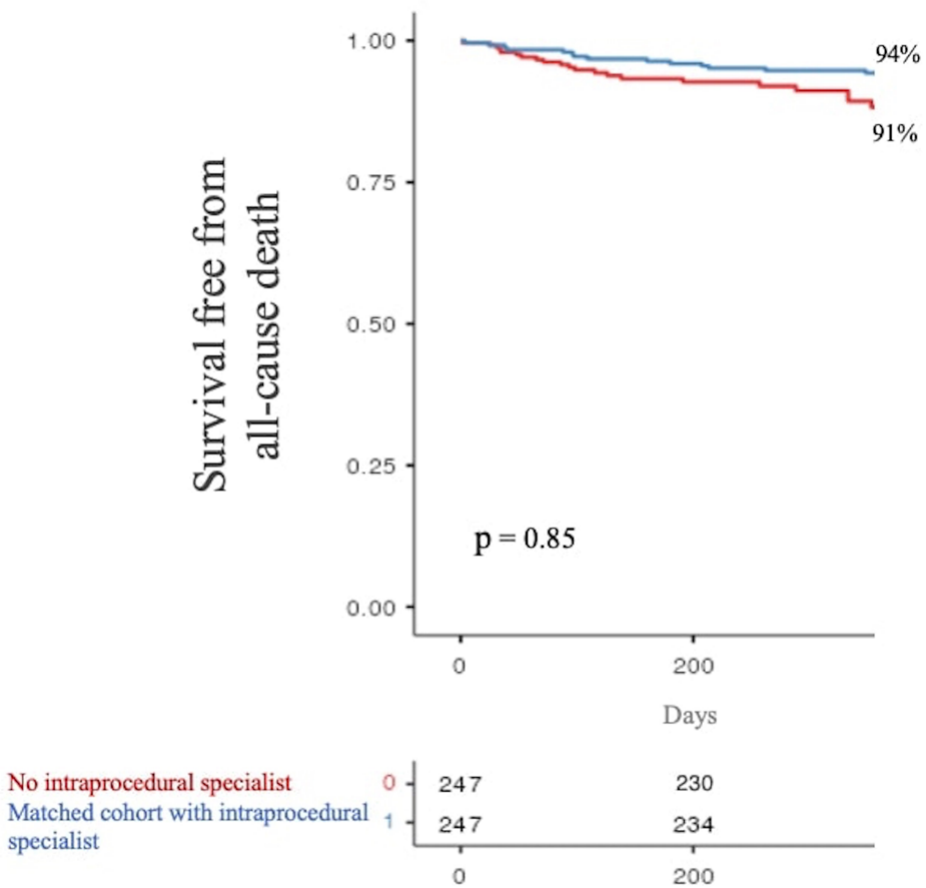
One-year survival free from all-cause death stratified by product specialist supervision during LAAO. LAAO, left atrial appendage occlusion.

At 1-year follow-up, the primary endpoint occurred in 21 patients (9%) of our baseline populations and in 14 patients (6%) of the matched cohort. Kaplan–Meier curves showed no significant difference in primary endpoint occurrence according to intraprocedural PS monitoring (*p* = 0.85, see Figure 2).

**Figure 3:**
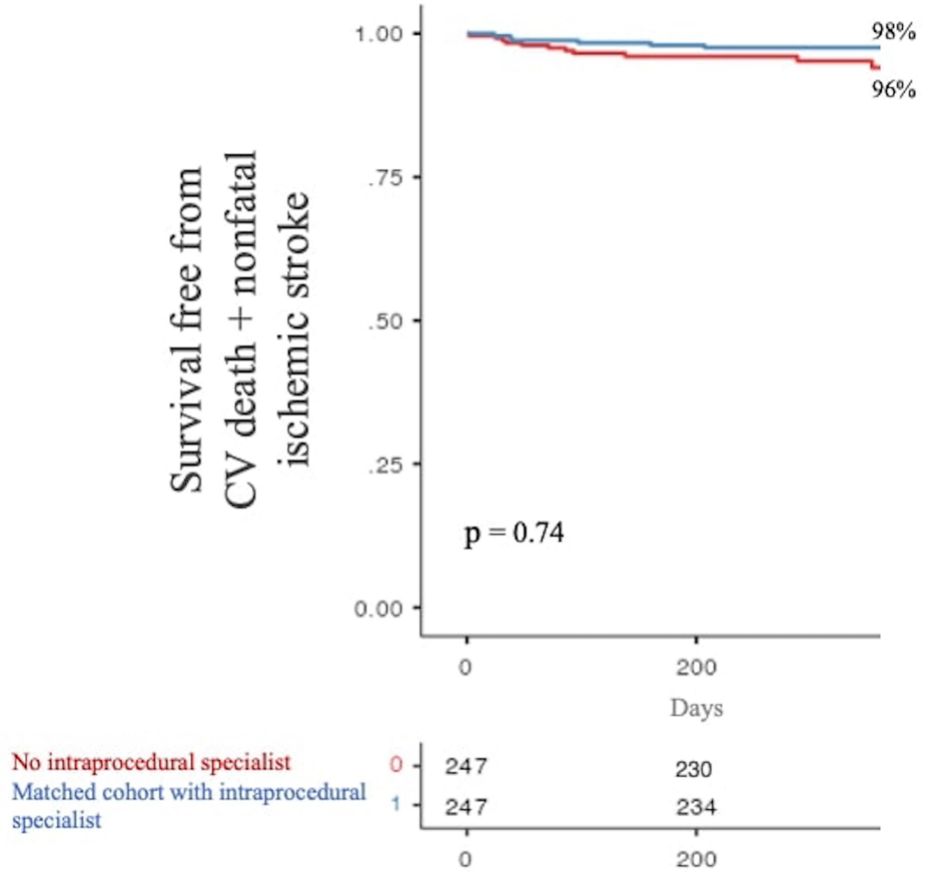
One-year survival free from the secondary composite endpoint stratified by product specialist supervision during LAAO. LAAO, left atrial appendage occlusion.

The secondary composite endpoint of CV mortality + nonfatal ischemic stroke at 1 year occurred in 11 (4%) and 6 (2%) patients without and with intraprocedural specialist supervision, respectively. Again, there was no significant difference in the occurrence of the secondary composite outcome between the two analyzed populations (*p* = 0.74, see Figure 3).

## Discussion

The main results of our study are as follows:

– This real-world experience from three high-volume centers shows that percutaneous LAAO seems to be a safe and effective procedure even in the absence of intraprocedural monitoring by the PS.
– No significant differences were found in term of procedural time, procedural success, and postprocedural AKI between our group and a matched population of patients who underwent LAAO with intraprocedural “typical” supervision.
– One-year survival free from ACD and from CV death + nonfatal ischemic stroke does not seem to differ between the two matched cohorts.

LAAO is a widespread thromboembolic preventive strategy in patients with AF and contraindication to long-term anticoagulant treatment. This approach proved to be non-inferior to oral anticoagulant therapies for stroke prevention with a prospect of lower bleedings rates on longer follow-up (2, 13–15).

Nonetheless, LAAO closure represents a complex procedure that may expose the patient to life-threatening complications such as major bleedings and cardiac tamponade; thus, it is usually performed by operators under the surveillance of the PS.

The 2016-SCAI/ACC/HRS Institutional and Operator Requirements for Left Atrial Appendage Occlusion document and the 2020-EHRA/EAPCI expert consensus statement on catheter-based LAAO specify the skills necessary for an operator to be authorized to perform this procedure; however, they do not report any recommendation about the need for intraprocedural specialist monitoring (16, 17). The role of the PS is of outmost importance, particularly in the context of interventionalist and echocardiographer without an extensive experience in the field of LAA closure. In fact, the PS may help the echocardiographer to appropriately measure the orifice, the landing zone, and the depth of the LAA, and to identify potential obstacle to the procedure, such as secondary lobes being too close to LAA orifice. Moreover, by suggesting fluoroscopic projections to best visualize LAA, he/she usually merges information from both the image technique in order to identify the most appropriate size of the device and of the access sheath to use. Finally, there is a significant contribution in transeptal puncture, device deployment, and, before its release, to ensure adequate positioning, compression, and stabilization. Of note, development of latest technologies, particularly in the context of remote-procedure monitoring, are allowing the PS to support both the interventionalist and the echocardiographer live in almost all the steps of the procedures; if these technologies will globally result with positive cost-effectiveness, the possibility to spread experience in LAAO procedures even to low-volume centers will further increase.

As COVID-19 pandemic has raised the issue of limited access of nonhospital staff to operative rooms, the push for operators to independently perform LAAO has recently increased. Accordingly, while the large majority of the procedures with PS support were performed before the COVID-19 pandemic, interventions without PS assistance were more frequently conducted after the pandemic breakout. In our experience, the first described in this setting to the best of our knowledge, procedural success was achieved in 98.4% of patients: a similar percentage is widely reported in the literature (varying from 88% to 98.8%) (17–19). Our rate of major complications too, namely, a 0.8% for procedural-related stroke and a 0.4% for hemodynamically relevant pericardial effusion, aligns with Watchman registries and randomized trials, which report a percentage ranging from 0% to 0.9% and from 0% to 4.1%, respectively (17–21). Although we experienced no periprocedural major bleedings, this finding strictly depends upon the definition used (in our case, type III–V according to the BARC classification).

Finally, even the mean CHA2DS2-VASc score of our population, 4, was similar to the mean baseline thromboembolic risk reported in most LAAO studies with Watchman device [mean CHA2DS2-VASc score from 2.2 to 4.5 (1, 2, 13, 18–21)].

Although during the last decade the number of LAAO procedures and, therefore, operator's expertise have dramatically increased, the similitude between our data and those reported in the literature may cautiously enforce our idea of LAAO feasibility in expert hands event without intraprocedural PS monitoring. A recent study examined the relationship between hospital procedural volume and in-hospital outcomes for patients undergoing percutaneous LAAO (22): higher procedural volume was associated with lower rates of in-hospital major adverse events (MAE), defined as a composite of mortality, stroke or transient ischemic attack, bleeding or transfusion, vascular complications, myocardial infarction, systemic embolization, and pericardial effusion or tamponade requiring pericardiocentesis or surgery. The inflection point of the significant volume-MAE association was visually estimated at 35 procedures/year (22). Although this retrospective study evaluated only in-hospital events, without long-term follow-up, it has enforced the call for the inclusion of a minimum institutional LAAO volume to ensure appropriate quality of care, and this concept is of particular relevance in the context of operators who may want to start performing percutaneous LAAO without intraprocedural PS support.

Specifically, when comparing our cohort with the non-supervised matched group, no significant difference was found in terms of procedural time, intraprocedural leak, cardiac tamponade, or procedure-related stroke. Only a single case of intraprocedural death (a cardiac tamponade complicated by cardiac arrest) was registered in our no-surveillance cohort. Moreover, a significant increase of periprocedural minor bleedings compensated by a reduced amount of major bleedings was noticed. As procedural time and intraprocedural complications did not differ between the two groups, one may argue that the opposed rate of bleedings may only be related to vascular access site or patient's intrinsic clinical problems.

A higher dosage of iodinated contrast medium was administered during procedures without specialist supervision, possibly due to the need of the operator to best characterize LAA anatomy and assess the correct device positioning before the release; however, the increased exposure to medium contrast did not lead to significant different rate of postprocedural AKI cases between the two groups.

The consistency of the intra- and periprocedural results is confirmed by 1-year follow-up outcomes, where all-cause mortality and the composed outcome of cardiovascular mortality and/or nonfatal ischemic stroke did not differ between the two groups, thus limiting the impact of intraprocedural PS monitoring on long-term “hard” clinical events.

### Study limitations

This study presents several limitations. First, this is an observational, retrospective study: therefore, it has the inherent limits of the study design, and our results must be confirmed in a larger sample size. Second, there may be an operative bias, as the procedures were performed in centers highly specialized in LAAO [now routinely performing >40 procedures per year (22)] with senior interventionalists presenting with different years of experience before undertaking these procedures without PS support and working in a strict collaboration along with senior intraprocedural echocardiographers. Therefore, these results may not be immediately reproducible in centers with lower experience in this field but should be cautiously interpreted as a potential novel operative strategy when performed in the appropriate intraprocedural context.

At follow-up, only a minority of patients underwent transesophageal study and, therefore, we could not rule out device-related thrombosis or significant paravalvular leak for the entire population; however, even in the eventuality of these complications, we did not find any difference in cardiovascular events at follow-up. We did not perform screening with neuroimaging examinations; therefore, we could not rule out differences in postprocedural silent cerebral ischemia. Because of the low incidence of intra- or postprocedural adverse events, independent predictors could not be searched. We cannot exclude that KM curves may significantly diverge with time, and longer clinical follow-up may be warranted to enforce our conclusions. Finally, we did not include antithrombotic therapy as a covariate in the propensity model as there were too many different regimens and we could not prove information regarding differences in antithrombotic therapies at follow-up.

## Conclusions

In conclusion, LAAO performed in high-volume centers without the surveillance of the PS is a feasible procedure that, when compared to matched populations that underwent LAAO with intraprocedural monitoring, did not result in lower rates of procedural success or higher rates of periprocedural complications, 1-year all-cause death, and cardiovascular death or nonfatal stroke.

## Data Availability

The data analyzed in this study is subject to the following licenses/restrictions: Data are available upon specific request to the corresponding author. Requests to access these datasets should be directed to margonato.davide@hsr.it.
